# Mental health and academic performance: a study on selection and causation effects from childhood to early adulthood

**DOI:** 10.1007/s00127-020-01934-5

**Published:** 2020-08-19

**Authors:** Sara Agnafors, Mimmi Barmark, Gunilla Sydsjö

**Affiliations:** 1 Division of Children’s and Women’s Health, Department of Biomedical and Clinical Sciences, Linköping University, 581 85 Linköping, Sweden; 2 Department of Sociology, Lund University, 221 00 Lund, Sweden

**Keywords:** Children, Education, Mental health, Socio-economic status, Social selection, Social causation

## Abstract

**Purpose:**

An inverse relationship between mental health problems and academic achievement is a well-known phenomenon in the scientific literature. However, how and when this association develops is not fully understood and there is a lack of longitudinal, population-based studies on young children. Early intervention is important if associations are to be found already during childhood. The aim of the present study was to investigate the development of the association between mental health and academic performance during different developmental periods of childhood and adolescence.

**Methods:**

Data from a longitudinal birth cohort study of 1700 children were used. Child mental health was assessed through mother’s reports at age 3, and self-reports at age 12 and 20. Academic performance was assessed through teacher reports on educational results at age 12 and final grades from compulsory school (age 15–16) and upper secondary school (age 18–19). The association between mental health and academic performance was assessed through regression models.

**Results:**

The results indicate that social selection mechanisms are present in all three periods studied. Behavioral and emotional problems at age 3 were associated with performing below grade at age 12. Similarly, mental health problems at age 12 were associated with lack of complete final grades from compulsory school and non-eligibility to higher education. Academic performance at ages 15 and 19 did not increase the risk for mental health problems at age 20.

**Conclusion:**

Mental health problems in early childhood and adolescence increase the risk for poor academic performance, indicating the need for awareness and treatment to provide fair opportunities to education.

**Electronic supplementary material:**

The online version of this article (10.1007/s00127-020-01934-5) contains supplementary material, which is available to authorized users.

## Introduction

An inverse relationship between mental health and educational attainment is a well-known phenomenon in the scientific literature of sociology, epidemiology and social psychiatry [1–3]. Despite nearly a century of research into the matter, no consensus has been reached about how the association develops and persists. Several studies have demonstrated the effect of educational attainment on mental health (social causation) [1, 3, 4] and likewise, there is support for the influence of mental health problems on educational attainment (social selection) [1, 2, 5]. Naturally, this knowledge comes mostly from research on the adult population as the level of SES and educational attainment are established over the years. However, there are several reasons to investigate the association between mental health and academic performance already during childhood. Social causation processes during childhood are dependent on the level of SES and educational tradition of the family of origin. Equally important, schooling is a central part of all of childhood, with the educational path starting at an early age. If an association between mental health and academic performance can be found already during childhood and adolescence, early recognition and interventions are warranted. However, there are few longitudinal, population-based studies on children and adolescents. As academic performance is associated with future educational attainment [6], and mental health problems during childhood increases the risk for subsequent mental health problems [7], early intervention is highly valuable. Given the multifactorial etiology of mental illness, and the number of factors predicting educational attainment, there is reason to assume that the relation between the two is complex [8].

Studies on the impact of educational achievement and academic performance on mental health in the younger population mainly include adolescents. In a meta-analysis of 17 original works, early school drop-out was found to be associated with substance abuse, depression and externalizing problems [4]. Moreover, academic performance in adolescence has been associated with suicide in men but not in women [9]. In a recent Swedish study, a low Grade Point Average (GPA) at age 16 was found to be associated with depression in early adulthood, and this association was attenuated by externalizing comorbidity [10]. In a large population-based cohort, Jonsson et al. [11] found that a low GPA was associated with hospitalization due to depression in adolescence. However, academic performance was assessed at age 16 and depression between ages 12 and 17 making a definite conclusion about the direction of the association precarious. With regard to younger children, Deighton et al. [12] found support for the effect of poor academic performance on subsequent internalizing problems in middle childhood. In sum, a couple of studies investigated the effect of academic performance on mental health in adolescence and early adulthood, although most studies focused mainly on depression.

Several population-based studies investigated the effect of mental health on academic performance in children and adolescents. In a longitudinal study, Fletcher showed that adolescent depression was linked to years of schooling, controlling for psychiatric comorbidity and sociodemographic factors [5]. In a study on 800 children followed from age 6 to 18, externalizing but not internalizing problems predicted poor academic performance [13]. Breslau and colleagues found that attention problems at age 6 predicted math and reading achievement at age 17, while no effect was seen for externalizing and internalizing problems [14]. In a cohort study of 400 children, McLeod and Kaiser found that internalizing and externalizing problems at age 6–8 strongly diminished the chance of accomplishing a high-school degree [15]. Deighton et al. [12] investigated the association between internalizing and externalizing problems and academic performance during middle childhood and early adolescence, confirming the effect of externalizing problems on later academic achievement. In a longitudinal population-based study, Miech et al. [8] found that externalizing problems had direct negative effects on adolescent school performance, while neither causation nor selection processes applied for depression and SES. The authors conclude that disorder-specific models are required. In sum, previous research shows that mental health problems predict academic performance from middle childhood and up to adulthood. Results seem, however, to vary by the type of mental health problems and assessments of whether mental health problems as early as pre-school age predict educational outcomes have not yet been done.

The present study uses a sample from a longitudinal cohort study to investigate whether mental health predicts academic performance and, vice versa, whether academic performance predicts mental health during different developmental periods in childhood and adolescence. The study offers the opportunity to control for a variety of variables potentially impacting mental health and academic performance, including maternal mental health and parental education level. Given the evidence presented above, it is likely that there are mechanisms in both directions but that these vary between populations, national/cultural contexts, age groups, types of mental illness, different aspects of social class/educational achievement, etc. Further studies are, therefore, warranted.

### Aim

The aim of the present study was to investigate the development of the association between mental health and academic performance during different developmental periods in childhood and adolescence. The following five hypotheses are used to test the associations empirically:

1. Internalizing and/or externalizing problems at age 3 increase the risk for poor academic performance at age 12.
2. Internalizing and/or externalizing problems at age 12 increase the risk for incomplete final grades from compulsory school (age 15–16).
3. Internalizing and/or externalizing problems at age 12 increase the risk for non-eligibility to higher education (age 18–19).
4. Incomplete final grades from compulsory school (age 15–16) increase the risk for internalizing and/or externalizing problems at age 20.
5. Non-eligibility to higher education increases the risk for internalizing and/or externalizing problems at age 20.

## Methods

### Subjects and procedures

**Fig. 1:**
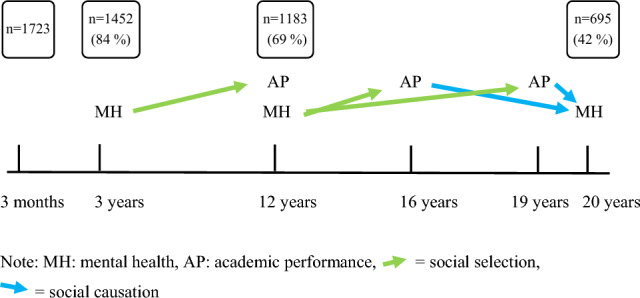
Model of the study outline and participants

Data from a longitudinal birth cohort study, the SESBiC study, were used [16]. All women who gave birth to children during 20 consecutive months 1995–1996 in five geographically adjacent municipalities in southern Sweden were asked to take part in the overarching study. Of those, 88% (*n* = 1723) accepted participation. For an overview of the study population and the waves of data collection, see Fig. 1. Among participating children, 52.8% were boys, and there were 27 pairs of twins. The baseline study and the 3-year follow-up were conducted at Child Welfare Centers (CWC’s), in connection with the routine age-based examination. At baseline, the mothers were interviewed by a psychologist, and at the 3-year follow-up, they filled out questionnaires on mental health and well-being for themselves as well as for their children.

The 12-year follow-up, was carried out at school as most of the children still lived in the catchment area. Research assistants supervised the children as they filled out questionnaires on mental health and well-being and helped with questions. A package of questionnaires including standardized instruments regarding mental health and well-being was sent to parents by ground mail. Teachers rated academic performance for reading, mathematics and English language respectively at the 12-year follow-up. At the 20-year follow-up, the now young adults answered standardized instruments on mental health and behavior. Parents had to give written consent for the child to be enrolled in the baseline, 3-year and 12-year follow-ups. Written consent was obtained from the young adults themselves at the 20-year follow-up.

### Instruments

The Edinburgh Postnatal Depression Scale (EPDS) [17] is a self-report scale designed to screen for postnatal depression in community samples. It holds 10 items ranged 0–3, with a total score of 30, a high score indicating postnatal depression. The EPDS refers to symptoms of depression and anxiety perceived during the recent week and was filled out by the mothers at baseline. A cut-off of 10 was set for the EPDS, which has been done previously for screening purposes [18]. In the analysis, the variable was, thus, dichotomized and women with a score of ≥ 10 were compared to women with a score of 9 or lower (reference).

The Child Behavior Checklist/2–3 (CBCL) [19] is a well-known form assessing child behavior into two main domains of internalizing and externalizing problems. The form holds 100 items, each scored 0–2 from “not a problem” to “often a problem”. The CBCL has been used previously in Scandinavian population-based studies and has shown good precision when screening for child psychiatric disorders [20]. The mothers filled out the Swedish version of the CBCL 2/3 at the 3-year follow-up. In logistic regression, the 90th percentile was used as cut-off. The variable was, thus, dichotomized, and children with a score within the 90th percentile were compared to children with a lower score (reference).

The Strengths and Difficulties Questionnaire (SDQ) is a screening instrument [21] consisting of 25 items divided between four problem subscales (emotional-, conduct-, hyperactivity- and peer problems) and one strength subscale (prosocial behavior). The self-report version was filled out by the children at the 12-year follow-up. In logistic regression, the 90th percentile was used as cut-off. The variable was, thus, dichotomized, and children with a score within the 90th percentile were compared to children with a lower score (reference).

The Symptom Checklist (SCL-25) was completed by the mothers at the 12-year follow-up [22]. The form consists of 25 items scoring on a scale of 1–4, from “not at all” to “extremely” and is designed to measure anxiety and depression during the most recent 14 days. When used in logistic regression, a cut-off was set to mean value 1.75 which has been used previously [23].

The teacher’s report form (TRF) [24] is a screening instrument for child behavior problems including information on academic performance. The TRF 5–18 was answered by the teachers at the 12-year follow-up and in this study, only information on child performance in reading, mathematics and English language was used. English is the secondary language for the study participants, taught from age 10 at the latest. The teachers rated child performance on a 5-point scale as “far below grade”, “somewhat below grade”, “at grade level”, “somewhat above grade” and “far above grade”. In models using logistic regression, the variable was dichotomized and “far below grade” and “somewhat below grade” was compared to “at grade level”, “somewhat above grade” and “far above grade” (reference).

The Adult Self Report [25] is a 126-item form assessing mental health divided into the two main domains of internalizing and externalizing problems. Each item is rated on a 3-point scale from “not true” to “somewhat or sometimes true” and “very true or often true”. The ASR has shown good validity and has been used previously in population-based studies [26]. The ASR was answered by the young adults at the 20-year follow-up.

### Register data

The Swedish school system is based on 10 years of compulsory education, followed by 3-year optional upper secondary education. Individuals without complete grades from upper secondary education may attend municipal adult education, with the possibility to achieve the eligibility requirements for university studies. In 2019, 84.3% of students received complete final grades from compulsory school [27], and approximately 80% completed upper secondary education. Final grades from compulsory and upper secondary school were obtained from the national statistics office. Lack of complete grades (and thereby inability to continue upper secondary school) was compared to complete final grades from compulsory school (reference). Non-eligibility to university studies/higher education was compared to eligibility to higher education (reference).

### Socio-demographic factors

Parental immigration status was noted at the baseline study, and children of one or both parents born abroad were compared to children of parents born in Sweden (reference). Information on school drop-out of the mothers was obtained at the baseline survey. Mothers who did not complete compulsory school or upper secondary school were compared to mothers who did (reference). Information on parental education was obtained at the 12-year follow-up. Mothers and fathers reported their highest level of education, and compulsory/upper secondary school (≤ 12 years of schooling) was compared to post-secondary education (> 12 years of schooling, reference level), based on the parent with the highest level of education.

### Data analysis

For frequencies of included variables, that is, mental health parameters (CBCL, SDQ, ASR), academic performance (performance in reading, mathematics and English language at age 12, final grades from compulsory school and upper secondary school) and control variables (maternal school drop-out, maternal mental health, parental education level, gender and parental immigrations status), see Table 1. Missing data ranged from 0% (gender) to 58.4% (ASR externalizing). To test the hypotheses of an association between mental health and education, bivariate linear regression or logistic regression was performed in five separate models. Linear regression was used for continuous dependent variables, and logistic regression was used for binary outcome variables. Then, for each model, multivariate linear or logistic regression was carried out controlling for maternal school drop-out, maternal mental health, parental education level, gender and parental immigration status, and when possible also for mental health at a previous data collection point. Stepwise regression was performed, excluding the control variable with the highest *p* value until all remaining control variables showed statistical significance. Gender was, however, included in all final models. In the linear regression tables the beta coefficients (*B*) and the 95% confidence intervals (CI) are presented, and in the logistic regression tables the odds ratios (OR) and their respective CI are presented. Statistical significance was defined as (two-sided) *p *≤ 0.05. All statistical analyses were performed using IBM SPSS version 24. For an overview of the study outline, see Fig. 1.

**Table 1:** Frequency characteristics of the study population

Variable	*N* (%)	Median/range
Baseline		
Gender	1723	
Girl	814 (47.2%)	
Parental immigration status	1704	
One parent born abroad	243 (14.3%)	
Maternal school drop-out at baseline	1626	
Yes	157 (9.7%)	
Maternal symptoms of PPD	1679	4/0–23
EPDS ≥ 10	204 (12.0%)	
3-year follow-up		
Behavioral problems	1428	7/0–34
CBCL externalizing ≥ 90th percentile	134 (9.4%)	
Emotional problems	1428	3/0–32
CBCL internalizing ≥ 90th percentile	138 (9.5%)	
12-year follow-up		
Mathematics performance	964	
Below grade	166 (17.2%)	
Reading performance	983	
Below grade	181 (18.4%)	
English language performance	960	
Below grade	200 (20.8%)	
Behavioral problems	1183	1/0–9
SDQ conduct ≥ 90th percentile	116 (9.8%)	
Emotional problems	1183	2/0–9
SDQ emotion ≥ 90th percentile	89 (7.5%)	
Maternal symptoms of depression	885	33/25–94
SCL25 ≥ M 1.75	161 (18.2%)	
Parental education level	919	
> 12 years of schooling	402 (43.7%)	
Age 16		
Compulsory school grades	1668	
Incomplete	183 (11%)	
Age 19		
Eligibility to higher education	1314	
Non-eligibility	297 (22.6%)	
20-year follow-up		
Behavioral problems	693	9/0–55
ASR externalizing ≥ 90th percentile	65 (9.4%)	
Emotional problems	695	11/0–57
ASR internalizing ≥ 90th percentile	68 (9.8%)	

*PPD* Postpartum depression, *EPDS* Edinburgh Postnatal Depression Scale, *CBCL* Child Behaviour Checklist, *SDQ* Strengths and Difficulties Questionnaire, *SCL25* Symptom Checklist 25 *ASR* Adult Self Report

### Drop-out rate analysis

At the 3-year follow-up, the retention rate was 84.2% (*n* = 1452). At the 12-year follow-up, the corresponding number was 68.7% (*n* = 1183). At the 20-year follow-up, two individuals had died, 10 had moved out of the country, 25 had incorrect or missing postal addresses, 19 individuals declined participation due to learning disabilities or autism resulting in 1667 eligible participants out of whom 41.7% (*n* = 695) accepted participation. For detailed information on the drop-out rate analysis, see Supplementary Material.

## Results

### Model 1, social selection

In bivariate analysis, externalizing problems at age 3 increased the risk for performing below grade in English and mathematics, but not reading, at age 12 (Table 2). Internalizing problems increased the risk for low performance in reading, English and mathematics (Table 2).

**Table 2:** Social selection

	OR (95.0% CI)	*p*-value	aOR (95.0% CI)	*p*-value	*N*
Model 1					
Reading 12 years below grade					
Internalizing 3 years ≥ 90th percentile	1.85 (1.09–3.13)	0.022	1.81 (0.96–3.43)	0.069	706
Gender			0.47 (0.31–0.71)	< 0.001	
Parental education level 12 years			0.44 (0.28–0.68)	< 0.001	
Externalizing 3 years ≥ 90th percentile	1.57 (0.91–2.68)	0.104	1.20 (0.62–2.34)	0.586	706
Gender			0.47 (0.31–0.72)	< 0.001	
Parental education level 12 years			0.44 (0.28–0.69)	< 0.001	
English language 12 years below grade					
Internalizing 3 years ≥ 90th percentile	1.88 (1.11–3.17)	0.018	2.15 (1.17–3.95)	0.013	688
Gender			0.93 (0.63–1.38)	0.721	
Parental education level 12 years			0.38 (0.25–0.58)	< 0.001	
Externalizing 3 years ≥ 90th percentile	2.37 (1.42–3.94)	0.001	2.65 (1.47–4.79)	< 0.001	688
Gender			0.96 (0.65–1.42)	0.837	
Parental education level 12 years			0.40 (0.26–0.61)	< 0.001	
Mathematics 12 years below grade					
Internalizing 3 years ≥ 90th percentile	1.89 (1.11–3.23)	0.020	1.92 (1.04–3.56)	0.038	695
Gender			0.98 (0.65–1.47)	0.926	
Parental education level 12 years			0.49 (0.32–0.76)	0.001	
Externalizing 3 years ≥ 90th percentile	1.86 (1.09–3.17)	0.023	1.43 (0.75–2.72)	0.273	695
Gender			1.00 (0.66–1.49)	0.979	
Parental education level 12 years			0.50 (0.32–0.77)	0.001	
Model 2					
Incomplete grades compulsory school					
SDQ emotion 12 years≥ 90th percentile	1.51 (0.77–2.93)	0.229	2.35 (1.07–5.12)	0.032	901
Gender			0.82 (0.48–1.42)	0.477	
Parental education level 12 years			0.34 (0.18–0.63)	0.001	
SDQ conduct 12 years≥ 90th percentile	2.42 (1.42–4.11)	0.001	2.60 (1.30–5.22)	0.007	901
Gender			1.00 (0.58–1.73)	0.990	
Parental education level 12 years			0.34 (0.18–0.64)	0.001	
Model 3					
Non-eligibility higher education					
SDQ emotion 12 years≥ 90th percentile	1.81 (1.06–3.09)	0.031	1.95 (1.06–3.59)	0.031	760
Gender			0.51 (0.36–0.74)	< 0.001	
Parental education level 12 years			0.36 (0.25–0.52)	< 0.001	
SDQ conduct 12 years≥ 90th percentile	2.53 (1.57–4.07)	< 0.001	2.24 (1.29–3.89)	0.004	760
Gender			0.57 (0.40–0.82)	0.002	
Parental education level 12 years			0.35 (0.24–0.52)	< 0.001	

Odds ratios in predicting academic performance

Bivariate and multivariate logistic regression. 0 is used as a reference level. *N* presented for adjusted models. Model 1: Dependent variables: academic performance in reading, English language and mathematics (0 = “at grade level”, “somewhat above grade “and “far above grade”, 1 = far below grade”, “somewhat below grade”). Independent variables: CBCL internalizing and externalizing (0 < 90th percentile, 1 ≥ 90th percentile), gender (0 = boys, 1 = girls), parental education level (0 ≤ 12 years of schooling, 1 > 12 years of schooling). Model 2: Dependent variables: final grades compulsory school (0 = complete grades, 1 = incomplete grades). Independent variables: SDQ emotion and conduct (0 < 90th percentile, 1 ≥ 90th percentile) gender (0 = boys, 1 = girls), parental education level (0 ≤ 12 years of schooling, 1 > 12 years of schooling). Model 3: Dependent variables: non-eligibility to higher education (0 = eligibility, 1 = non-eligibility). Independent variables: SDQ emotion and conduct (0 < 90th percentile, 1 ≥ 90th percentile) gender (0 = boys, 1 = girls), parental education level (0 ≤ 12 years of schooling, 1 > 12 years of schooling)

*CI* confidence interval, *OR* odds ratio, *CBCL* Child Behaviour Checklist, *SDQ* Strengths and Difficulties Questionnaire

In multivariate analysis, externalizing problems at age 3 were still associated with English language performance at age 12, after controlling for parental education level and gender (Table 2). When including concurrent conduct problems in the model, the association remained. No associations were seen for externalizing problems at age 3 on mathematics and reading performance at age 12 (Table 2). Internalizing problems at age 3 were shown to predict performing below grade in English language and mathematics after controlling for parental education level and gender (Table 2). The associations remained even when including concurrent emotional problems in the models.

### Model 2, social selection

In bivariate analysis, conduct problems at age 12 was associated with lack of final grades from compulsory school (Table 2). No association between emotional problems at age 12 and incomplete grades from compulsory school was found (Table 2).

In multivariate analysis, conduct problems at age 12 predicted incomplete grades from compulsory school after controlling for gender and parental education level at the child’s age 12 (Table 2). Likewise, an association was found between internalizing problems at age 12 and incomplete grades from compulsory school after controlling for the above-mentioned variables (Table 2).

### Model 3, social selection

In bivariate analysis, both conduct and emotional problems at age 12 were associated with non-eligibility to higher education (Table 2).

In multivariate analysis, conduct problems at age 12 increased the risk for non-eligibility to higher education after controlling for gender and parental education level at the child’s age 12 (Table 2). The same applied for emotional problems.

### Model 4, social causation

In bivariate analysis, incomplete grades from compulsory school were associated with externalizing problems at age 20, while no association was found for internalizing problems (Table 3).

**Table 3:** Social causation

	*B* (95.0% CI)	*p*-value	Adjusted *B* (95.0% CI)	*p*-value	*N*
Model 4					
ASR internalizing 20 years					
Compulsory school grades	3.24 (− 0.16–6.65)	0.062	2.56 (− 0.54–6.67)	0.220	560
Gender			5.38 (3.62–7.14)	< 0.001	
EPDS (maternal, baseline)			0.38 (0.14–0.61)	0.002	
SDQ emotion 12 years			1.27 (0.84–1.72)	< 0.001	
ASR externalizing 20 years					
Compulsory school grades	3.24 (0.83–5.65)	0.009	0.81 (− 2.21–3.83)	0.600	551
Gender			1.05 (− 0.16–2.25)	0.088	
EPDS (maternal, baseline)			0.21 (0.05–0.37)	0.009	
SDQ conduct 12 years			0.84 (0.39–1.30)	< 0.001	
SDQ emotion 12 years			0.39 (0.07–0.71)	0.015	
Model 5					
ASR internalizing 20 years					
Eligibility higher education	0.04 (− 2.12–2.20)	0.970	− 0.44 (− 2.68–1.79)	0.698	503
Gender			5.61 (3.83–7.39)	< 0.001	
EPDS (maternal, baseline)			0.32 (0.08–0.56)	0.009	
SDQ emotion 12 years			1.24 (0.80–1.69)	< 0.001	
ASR externalizing 20 years					
Eligibility higher education	1.51 (0.08–2.94)	0.038	0.55 (− 0.94–2.05)	0.467	498
Gender			0.80 (− 0.43–2.02)	0.201	
EPDS (maternal, baseline)			0.22 (0.06–0.38)	0.008	
SDQ conduct 12 years			0.71 (0.24–1.19)	0.003	
SDQ emotion 12 years			0.45 (0.12–0.78)	0.007	

Bivariate and multivariate linear regression predicting mental health

Bivariate and multivariate linear regression. *N* presented for adjusted models. Model 4: Dependent variables: ASR internalizing and externalizing scores. Independent variables: final grades compulsory school (0 = complete grades, 1 = incomplete grades), gender (0 = boys, 1 = girls), EPDS total score, SDQ emotion and conduct subscales. Model 5: Dependent variables: ASR internalizing and externalizing scores. Independent variables: Eligibility to higher education (0 = eligibility, 1 = non-eligibility), gender (0 = boys, 1 = girls), EPDS total score, SDQ emotion and conduct subscales

*CI* confidence interval, *OR* odds ratio, *EPDS* Edinburgh Postnatal Depression Scale, *SDQ* Strengths and Difficulties Questionnaire, *ASR* Adult Self Report

In multivariate analysis, no association was found between incomplete grades from compulsory school and externalizing problems at age 20 after controlling for gender, maternal symptoms of postpartum depression, conduct problems at age 12 and emotional problems at age 12 (Table 3). However, when controlling only for gender and maternal symptoms of postpartum depression, incomplete grades from compulsory school increased the risk for externalizing problems at age 20 (OR 2.77, CI 0.32–5.22). No association was found between incomplete grades from compulsory school and internalizing problems at age 20 after controlling for gender, maternal symptoms of postpartum depression and emotional problems at age 12 (Table 3).

### Model 5, social causation

Non-eligibility to higher education was associated with externalizing problems at age 20 in bivariate analysis (Table 3). No association was found for internalizing problems.

In multivariate analysis, no association was found between non-eligibility to higher education and externalizing problems at age 20, controlling for gender, maternal symptoms of postpartum depression, conduct problems at age 12 and emotional problems at age 12 (Table 3). Likewise, no association was found between non-eligibility to higher education and internalizing problems at age 20 controlling for above mentioned factors (Table 3).

To further investigate the support for the social causation theory using data from the SESBiC study, whether academic performance at age 12 predicted internalizing and externalizing problems at age 20 was investigated; however, no associations were found. Likewise, parental education level at the child’s age 12 did not predict internalizing or externalizing problems at age 20.

## Discussion

The aim of the present study was to investigate whether mental health predicts academic performance and, vice versa, whether academic performance predicts mental health during different developmental periods in childhood and adolescence. The main findings are discussed below.

Externalizing problems at age 3 predicted academic performance (English language) at age 12, after controlling for a number of relevant factors. Conduct problems at age 12 were also found to increase the risk for incomplete grades from compulsory school and non-eligibility for higher education. Additionally, externalizing problems at age 3 increased the risk for incomplete final grades from compulsory school (data not shown). These findings are in line with previous studies investigating the association between externalizing problems during middle childhood and adolescence and subsequent academic achievement [8, 14, 15]. Externalizing problems include Conduct Disorder and Oppositional Defiant Disorder which implicates behavioral disturbances that could affect the adjustability in the classroom, and thereby the performance. Early behavioral problems might also trigger child–teacher conflicts and social exclusion leading to negative experiences of the school environment.

In the present study, selection effects were also found for internalizing problems on academic achievement. Mental health at pre-school age was associated with academic performance (English and mathematics) at age 12. Similarly, internalizing problems at age 12 increased the risk for incomplete grades at ages 15 and 19. Previous research shows, however, mixed results. A recent study found strong selection effects for internalizing problems such as anxiety and depression in an adult twin-population [2]. Internalizing problems in early school age has been shown to diminish the chances of completing a high-school degree [15]. In contrast, other studies found no effects of internalizing problems on academic performance from childhood to adolescence [13], or from adolescence to early adulthood [8]. With the background of previous conflicting results, the present study adds to the literature confirming social selection processes for the association between internalizing problems and academic performance.

Interestingly, boys were more likely to perform below grade in reading at age 12 and to be non-eligible for higher education compared to girls. No gender differences were noted for lack of compulsory school grades. In fact, the impact of gender on eligibility to higher education in the present study was considerable; 61% of non-eligible individuals were boys. Gender differences in academic performance with an advantage for girls are a well-known phenomenon [28].

Incomplete grades from compulsory school (age 15–16) were not associated with mental health at age 20. When controlling only for gender and maternal symptoms of postpartum depression, incomplete grades from compulsory school predicted externalizing problems at age 20. When controlling for mental health problems at age 12, however, the association diminishes, indicating that the association is a result of social selection rather than social causation mechanisms. Regarding the effect of academic performance on internalizing problems, no associations were found neither in bivariate nor in multivariate analysis.

These findings stand in contrast to the previous findings on the adult population including a meta-analysis, where the risk of reporting depression was almost doubled in low SES groups [29]. Similarly, Miech et al. found effects of adolescent school performance on anxiety conditions [8], while Sörberg Wallin et al. demonstrated an increased risk for depressive symptoms in early adulthood following poor academic performance in adolescence [10]. The social causation models in the present study were limited by a large drop-out rate, and a higher retention rate might have rendered other results. Also, at the age of 20, many individuals have not established a stable level of SES; university students not yet even graduated. For a child, poor academic performance does not have the same direct consequences as low educational attainment might in adulthood (possibly lower income, lesser ability to compete on the labor market). Moreover, the Swedish society offers additional possibilities to complete upper secondary education, which could influence the effect of poor academic performance on mental health during adolescence. Academic performance in childhood is, however, related to later educational attainment [6], which in turn has a strong effect on mental health in adulthood. It is, therefore, still, from a mental health perspective, of utterly importance that effort is being made to support children who perform “below grade level” to prevent health risks associated with low SES later in life.

Girls had a fivefold increased risk for internalizing problems at age 20 compared to boys; however, no gender differences were seen for externalizing problems. Previous results support gender differences in mental health with a pattern of higher frequency of internalizing problems in girls and higher prevalence of externalizing problems in boys [30]. In the present study, girls participated in the 20-year follow-up to a greater extent than boys (51.4% compared to 30.5%, *p* < 0.001), possibly impacting the results.

## Limitations

While the present study is strengthened by the large number of participants and the longitudinal design, a few limitations need to be considered when interpreting the results. First, using the social selection and social causation approach in a child population means that possible associations need to be interpreted carefully. While the social causation hypothesis generally implies that lower education level brings lower income, unhealthier life style and more life stressors, this does not readily apply during childhood and adolescence. Rather, children are under influence of the environmental circumstances impacting their parents and thus, a number of possibly confounding factors need to be considered. We have included several such factors in the models but cannot be certain that some important variable has not been left out. Second, a long period of time elapsed between the measures of mental health problems and academic performance, possibly diluting the effects. Narrower timespans between the follow-ups, or additional follow-ups would have been of great value. Moreover, while the influence of mental health on academic performance was modelled with three separate outcomes of academic performance during adolescence, the influence of academic performance on mental health was based on one single measurement of mental health at age 20. The effect of academic performance on mental health might have been stronger if measured for example within the year after receiving incomplete grades from compulsory school. Third, there was a considerable drop-out (58.3%) at the 20-year follow-up. In general, differences between participants and non-participants were noted on educational variables rather than mental health parameters. Moreover, fewer 20-year olds whose parents were born abroad and fewer men than women chose to take part in the 20-year follow-up. Since immigration status often is associated with lower SES [31], the skewed drop-out could have an impact on the results. However, parental immigration status was not found to be strongly associated with the outcomes measured in the present study. The skewness regarding men and women might have contributed to a lower degree of externalizing problems, as this is more common in men than in women. While no difference between participants and non-participants regarding mental health at age 3 or 12 was noted, we cannot completely rule out the possibility that individuals who developed mental health problems after 12 years of age were less likely to participate in the follow-up. If so, that would diminish the chance of detecting an association between academic performance in adolescence and mental health problems in early adulthood in line with the social causation hypothesis.

## Conclusions

The study adds to the existing literature by the use of a large, two-generational cohort, and longitudinal prospective design with multiple data collection points, investigating the association between mental health and academic performance during different developmental periods from age 12 to 20. The results emphasize the necessity to detect externalizing and internalizing problems at a young age and continuously throughout the school years. In practice, it means that these kinds of problems need to be noticed at preschool age and educational practices adjusted and adequate treatment given to promote transition to the school environment and completion of compulsory school. Knowledge and recognition of the potential effects of internalizing problems on academic performance might be especially important as these problems tend to be less explicit to others compared to externalizing problems.

No support was found for the association between academic performance during adolescence and mental health status in early adulthood in this Swedish context. However, this result needs to be interpreted in the light of a considerable drop-out rate and a long time span between measures of impact and outcome.

## Electronic supplementary material

Below is the link to the electronic supplementary material.

Supplementary material 1 (DOCX 14 kb)
