# Correction: Mental health and academic performance: a study on selection and causation effects from childhood to early adulthood

**DOI:** 10.1007/s00127-023-02560-7

**Published:** 2023-09-16

**Authors:** Sara Agnafors, Mimmi Barmark, Gunilla Sydsjö

**Affiliations:** 1 Division of Children’s and Women’s Health, Department of Biomedical and Clinical Sciences, Linköping University, 581 85 Linköping, Sweden; 2 Department of Sociology, Lund University, 221 00 Lund, Sweden

**Correction: Social Psychiatry and Psychiatric Epidemiology (2021) 56:857–866** 10.1007/s00127-020-01934-5

The abstract for this article was inadvertently truncated after the text 'Health'; the following text was missing ‘Problems’.

The correct sentence as follows,

“An inverse relationship between mental health problems and academic achievement is a well-known phenomenon in the scientific literature”.

The original article has been corrected.

