# Supplementary material for: Mental health and academic performance: a study on selection and causation effects from childhood to early adulthood
Source: Soc Psychiatry Psychiatr Epidemiol. 2020 Aug 19;56(5):857–66. doi: 10.1007/s00127-020-01934-5 (PMC8068628; doi:10.1007/s00127-020-01934-5)
Supplement: Supplementary file 1 — Supplementary material 1 (DOCX 14 kb) [file 127_2020_1934_MOESM1_ESM.docx]

**Supplementary material. Drop-out analysis. Participation rate at 20-year follow-up based on variables included in the study.**

| **Variable** |  | | **p** |
| --- | --- | --- | --- |
| **Baseline** |  |  |  |
| Gender female/male | 51.4% | 30.5% | <0.001 |
| Parental immigration status no/yes | 41.8% | 33.3% | 0.013 |
| Maternal school drop-out no/yes | 41.0% | 34.4% | 0.106 |
| Maternal symptoms of PPD no/yes | 41.5% | 33.8% | 0.037 |
| **3-year follow-up** |  |  |  |
| Behavioral problems (CBCL ext) no/yes | 43.3% | 38.8% | 0.311 |
| Emotional problems (CBCL int) no/yes | 43.0% | 41.9% | 0.801 |
| **12-year follow-up** |  |  |  |
| Mathematics performance average-above/low | 50.6% | 32.5% | <0.001 |
| Reading performance average-above/low | 50.9% | 33.7% | <0.001 |
| English language performance average-above/low | 49.1% | 41.0% | 0.042 |
| Behavioral problems (SDQ conduct) no/yes | 47.9% | 47.4% | 0.922 |
| Emotional problems (SDQ emotion) no/yes | 47.5% | 51.7% | 0.451 |
| Maternal symptoms of depression no/yes | 54.7% | 51.5% | 0.471 |
| Parental education level high/low | 59.5% | 45.5% | <0.001 |
| **Age 16** |  |  |  |
| Compulsory school grades yes/no | 43.5% | 23.0% | <0.001 |
| **Age 19** |  |  |  |
| Eligibility to higher education yes/no | 48.8% | 37.0% | <0.001 |

Note: PPD = Postpartum Depression, CBCL = Child Behaviour Checklist, SDQ = Strenghts and Difficulties Questionnaire.
